# Cutaneous Reactions Following Anticancer Drug Therapy in a Tertiary Care Centre

**DOI:** 10.31729/jnma.8416

**Published:** 2024-01-31

**Authors:** Deeptara Pathak Thapa, Sudip Shrestha, Saloni Shrestha

**Affiliations:** 1 Department of Dermatology, Nepal Medical College and Teaching Hospital, Jorpati, Kathmandu, Nepal; 2 Department of Medical Oncology, Nepal Cancer Hospital and Research Centre, Harisiddhi, Lalitpur, Nepal; 3 Nepal Medical College and Teaching Hospital, Jorpati, Kathmandu, Nepal

**Keywords:** *cancer*, *chemotherapy*, *drug side effects*, *skin*

## Abstract

**Introduction:**

Cutaneous reactions are dermatologica! abnormalities that can occur after anticancer drug therapy in cancer patients. Cutaneous reactions can range from mild dermatological disorders to life-threatening medical conditions and may worsen a patient's quality of life. This study aimed to find out the prevalence of cutaneous reactions following anticancer drug therapy in a tertiary care centre.

**Methods:**

A descriptive cross-sectional study was conducted among cancer patients following anticancer therapy in the outpatient department of dermatology of a tertiary care centre between 1 October 2021 to 30 December 2022. Convenience sampling was done. The point estimate was calculated at a 95% Confidence Interval.

**Results:**

Among 3,288 patients, the prevalence of cutaneous reactions following anticancer drug therapy was seen in 73 (2.22%) (1.72-2.72, 95% Confidence Interval). The mean age was found to be 49.42±1.45 years. Anagen effluvium was the frequently observed cutaneous reaction 22 (30.10%) followed by palmar-plantar erythrodysesthesia 14 (19.94%).

**Conclusions:**

The prevalence of cutaneous reactions following anticancer drug therapy among cancer patients was found to be lower as compared to the studies conducted in similar settings. An interdisciplinary approach is required to identify cutaneous reactions to anticancer therapy and to navigate change in the treatment plan.

## INTRODUCTION

Cutaneous reactions are dermatological abnormalities that can occur after systemic drug administration.^1^ Cytotoxic effects of anticancer drugs can affect the skin barrier and mucosa resulting in skin rashes, xerosis, hyperpigmentation and life-threatening medical conditions like Steven-Johnson syndrome and toxic epidermal necrolysis. It also affects hair and nails, leading to dermatological conditions such as alopecia, paronychia and melanonychia.^2^

Cutaneous reactions can be seen in patients receiving chemotherapy as a part of anticancer drug therapy in cancer patients and such reactions can worsen patients' quality of life.^3^ There are very few studies conducted in Nepal regarding cutaneous reactions following anticancer drug therapy.

This study aimed to find out the prevalence of cutaneous reactions following anticancer drug therapy in a tertiary care centre.

## METHODS

A descriptive cross-sectional study was conducted in the Outpatient Department of Dermatology at Nepal Cancer Hospital and Research Centre (NCHRC) from 1 October 2021 to 30 December 2022 after obtaining ethical approval from the Institutional Review Committee of the hospital (Reference number: IRCNCHRC/003/2077). All patients giving consent for history taking, physical examination and necessary investigations, and receiving anticancer drugs due to anticancer therapy within the study period were included in the study. Patients who were under radiotherapy and had cutaneous reactions unrelated to anticancer drug therapy were excluded from the study. Convenience sampling was done. The sample size was calculated using the following formula:


$$\begin{array}{ll} \text{n} & ={\text{Z}}^{2}\times \frac{\text{p}\times \text{q}}{{\text{e}}^{\text{2}}}\\ & =1.96^{2}\times \frac{0.048\times 0.952}{0.01^{2}}\\ & =1,755 \end{array}$$

Where,

n = minimum required sample size
Z = 1.96 at 95% Confidence Interval (CI)
p = prevalence taken as 4.8% from previous study ^4^
q = 1-p
e = margin of error, 1%

The minimum required sample size was 1,755. However, 3,288 patients were included in the study.

Data regarding, demographic variables, concomitant cancer, duration since diagnosis, chemotherapeutic agents administered with their dose, duration and dermatological manifestation due to drug reaction were noted in a proforma along with findings of a thorough detailed cutaneous examination of skin, hair, nail and mucosa.

Data was entered in Microsoft Excel 2019 and analyzed using IBM SPSS Statistics version 16.0. The point estimate was calculated at a 95% CI.

## RESULTS

Among 3,288 patients, the prevalence of cutaneous reactions following anticancer drug therapy was seen in 73 (2.22%) (1.72-2.72, 95% CI) patients. Anagen effluvium was the frequently observed cutaneous reaction 22 (30.10%) followed by palmar-plantar erythrodysesthesia 14 (19.94%), acneform lesions with seborrheic dermatitis 6 (9.06%), eczema 6 (9.06%) as shown in (Table 1).

**Table 1:** Types of cutaneous reactions following use of anticancer drugs (n= 73).

Cutaneous manifestation	n (%)
Anagen effluvium	22 (30.10)
Palmar-plantar erythrodysesthesia	14 (19.94)
Eczema	6 (9.06)
Acne with seborrheic dermatitis	6 (9.06)
Maculopapular rash	4 (5.48)
Xerosis with Anagen effluvium	3 (4.17)
Xerosis	3 (4.17)
Pruritus	3 (4.17)
Urticaria	2 (2.73)
Vasculitis	2 (2.73)
Others^*^	8 (11.12)

* Others: Melanonychia, Pseudocellulitis, Mucositis, Prurigo nodularis, P. folliculitis, lipodermatosclerosis, Photodermatitis, scalp folliculitis.

**Figure 1:**
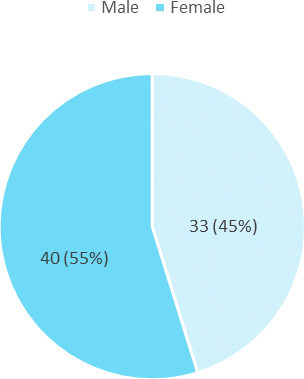
Gender-wise distribution (n= 73).

The prevalence of cutaneous reaction was seen more n females 40 (55%) (Figure 1).

The age range of presentation was (17 years-83 years) with a mean age of 49.42±1.45 years. The patients with age >45 developing cutaneous reactions were 38 (52.05%) (Table 2).

**Table 2:** Age-wise distribution (n= 73).

Age (years)	n (%)
<15	1 (1.37)
15-45	34 (46.57)
>45	38 (52.05)

Oxaliplatin 5-Fluorouracil 10 (13.70%) and Cisplatin+Gemcitabine 10 (13.70%) were the most commonly prescribed anticancer drug regimens in our study with cutaneous reactions (Table 3).

**Table 3:** Prescribed anticancer drug regimens in patients developing cutaneous reactions (n= 73).

Anticancer Drugs and Regimens	n (%)
Oxaliplatin+5-Fluorouracil	10 (13.69)
Cisplatin+Gemcitabine	10 (13.69)
Carboplatin+Paclitaxel	7 (9.59)
Doxorubicin+Cyclophosphamide	6 (8.22)
Doxorubicin + Cyclophosphamide + Etoposide	5 (6.85)
Carboplatin+Docetaxel	4 (5.48)
Cisplatin+5-Fluorouracil	3 (4.11)
Rituximab + Cyclophosphamide + Vincristine+Cyclophosphamide	3 (4.11)
Cisplatin	3 (4.11)
Carboplatin+Nanoxel	3 (4.11)
Carboplatin+Premetrexed	3 (4.11)
Vincristine+Cyclophosphamide	2 (2.74)
^†^Others	14 (19.18)

† Others: Rituximab+Cyclophosphamide+Vincristine+ Doxorubicin, Daunorubicin, Docetaxel + Ramucinamab, Osimertinib, Cituximab, Cituximab+Oxaliplatin+ 5-Fluorouracil, Pembrolizumab, Cyclophosphamide+5-Fluorouracil+Tamoxifen, Carboplatin+Docetaxel+Trastuzumab, Pablociclib, Bevacizumab, Bendamustine, Paclitaxel+Cisplatin, Transtazumab+paclitaxel.

## DISCUSSION

The hospital-based prevalence of cutaneous reactions to anticancer drugs in our study was (2.22%). A study done in Brazil by reviewing data from the medical record section showed the prevalence of cutaneous adverse events to be 18%,^5^ and another study from India showed 38.4% cutaneous adversities to chemotherapy.^3^ As compared to these studies, the prevalence of cutaneous reactions in our setting is lower, which might be due to a lower number of hospital visits or short periods of follow-up or the low number of reports of dermatological concerns or other unknown factors specific to our setting.

Different anticancer drugs like epidermal growth factor receptor (EGFR) inhibitors, taxanes, vinca alkaloids antimetabolites, genotoxic agents hydroxyurea, cyclophosphamide, doxorubicin, vincristine, prednisolone (CHOP) and ABVD (doxorubicin, bleomycin, vinblastine, dacarbazine) regimens are seen to be associated with dermatologic reactions.^6^ In our study we found female preponderance with 55%, similarly reported literature also found females as a majority with cutaneous reactions.^7-9^ The reason may be due to gender-wise differences in pharmacokinetics and pharmacodynamics along with hormonal changes which might alter the pharmacokinetics of drugs.^7^

The majority of the patients in our study were aged more than 45 years 38 (52.10%), which is lower than the past study.^7^ Therefore elderly patients should be monitored vigilantly with anticancer therapy. The most common dermatological condition in this study was melanonychia (78.7%) followed by anagen effluvium (37.7%) and acneiform rash (27.7%).^5^ Anagen effluvium 22 (30.10%) was the frequently observed cutaneous reaction in our study, similar findings were also seen in the studies in the literature.^3,6,9,10^ In our study it was commonly found to be associated with platinum- based regimens. Palmar-plantar erythrodysesthesia 14 (19.94%) also known as hand and foot syndrome was the second most common manifestation in our study. Similar findings were also seen in a previous study.^3,10^

Certain anticancer drugs inhibit both vascular endothelial growth factor receptor and platelet- derived growth which disrupts the normal repair process involving both capillaries and fibroblasts and leads to inflammation which presents as painful symmetric erythema over palms and soles and even blisters in severe cases.^2,5,6^ In our study we found common regimens associated with palmar-plantar erythrodysesthesia were Doxorubicin+cyclophosphamide, Carboplatin + Paclitaxel and Cisplatin+Gemcitabine. Acneform lesions with seborrheic dermatitis-like manifestation were seen in (9.06%) of patients in our study and were commonly seen with regimens with Trastuzumab+paclitaxel, Carboplatin+Docetaxel+Transtazumab and Bevacizumab. Targeted therapy agents like epidermal growth factor inhibitor(EGFR) trastuzumab, Cetuximab etc are associated with hyperkeratosis, follicular plugging and inflammation which leads to acne and seborrheic-like presentation.^11,12^ Eczema was observed in 6 (9.06%), commonly associated with the regimen, Doxorubicin+ cyclophosphamide+ Etoposide in our study, though it is commonly seen in spindle inhibitors like vinca alkaloids and taxanes.^2^ In our study, xerosis and pruritus were seen in 4%, similar findings were also seen in studies conducted in the past.^3,9^ Urticaria and vasculitis were observed 2 (2.73%) each and were commonly seen with platinum-based regimens in our study. In a study, vasculitis was also seen in (<1%).^8^

Other cutaneous reactions like scalp folliculitis, photodermatitis, melanonychia, pseudocellulitis, pityriasporum folliculitis (P. folliculitis), mucositis, prurigo nodularis and lipodermatosclerosis were observed in 1 (1.39%) each in our study. The commonest regimens in our study having adverse cutaneous reactions were seen with Oxaliplatin+ 5-Fluorouracil 10 (13.70%) and Cisplatin+Gemcitabine 10 (13.70%). In a similar study, the most common regimen having cutaneous drug reactions was seen with cisplatin+paclitaxel and paclitaxel and carboplatin.^3^ The commonest drug in the regimens having adverse reactions were platinum-based drugs which were observed both in our study and also in a similar study.^3^

Though we have tried to be comprehensive in our evaluation of patients presenting to our hospital, we could have missed patients who did not report their dermatological problems or did not follow up during the study period. Besides this limitation, due to the cross-sectional nature of the current study, patients could not be followed up for a longer period of time or evaluated for the resolution of the dermatological manifestations following treatment. Since most anticancer drugs in the current study were administered in regimens, specific agents that caused side effects could not be identified.

## CONCLUSIONS

The prevalence of cutaneous reactions following anticancer drug therapy among cancer patients was found to be lower as compared to the studies conducted in similar settings. An interdisciplinary approach is required to identify cutaneous reactions to anticancer therapy and to navigate change in the treatment plan. Prospective studies with larger sample sizes are needed to know more about cutaneous reactions of specific drug therapy. Follow-up of cancer patients along with awareness programs targeting physicians and the public are warranted.
